# Capture of carbon dioxide and hydrogen by engineered *Escherichia coli*: hydrogen-dependent CO_2_ reduction to formate

**DOI:** 10.1007/s00253-021-11463-z

**Published:** 2021-07-31

**Authors:** Felix Leo, Fabian M. Schwarz, Kai Schuchmann, Volker Müller

**Affiliations:** Molecular Microbiology & Bioenergetics, Institute of Molecular Biosciences, Johann Wolfgang Goethe University, Max-von-Laue-Str. 9, 60439 Frankfurt am Main, Germany

**Keywords:** Biocatalysis, Heterologous enzyme production, Hydrogen-dependent CO_2_ reductase, *Acetobacterium woodii*, CO_2_ capture

## Abstract

**Abstract:**

In times of global climate change and the fear of dwindling resources, we are facing different considerable challenges such as the replacement of fossil fuel–based energy carriers with the coincident maintenance of the increasing energy supply of our growing world population. Therefore, CO_2_ capturing and H_2_ storing solutions are urgently needed. In this study, we demonstrate the production of a functional and biotechnological interesting enzyme complex from acetogenic bacteria, the hydrogen-dependent CO_2_ reductase (HDCR), in the well-known model organism * Escherichia coli*. We identified the metabolic bottlenecks of the host organisms for the production of the HDCR enzyme complex. Here we show that the recombinant expression of a heterologous enzyme complex transforms *E. coli* into a whole-cell biocatalyst for hydrogen-driven CO_2_ reduction to formate without the need of any external co-factors or endogenous enzymes in the reaction process. This shifts the industrial platform organism *E. coli* more and more into the focus as biocatalyst for CO_2_-capturing and H_2_-storage.

**Key points:**

• *A functional HDCR enzyme complex was heterologously produced in E. coli.*

• *The metabolic bottlenecks for HDCR production were identified.*

• *HDCR enabled E. coli cell to capture and store H*_*2*_ *and CO*_*2*_ *in the form of formate.*

**Supplementary Information:**

The online version contains supplementary material available at 10.1007/s00253-021-11463-z.

## Introduction

**Fig. 1:**
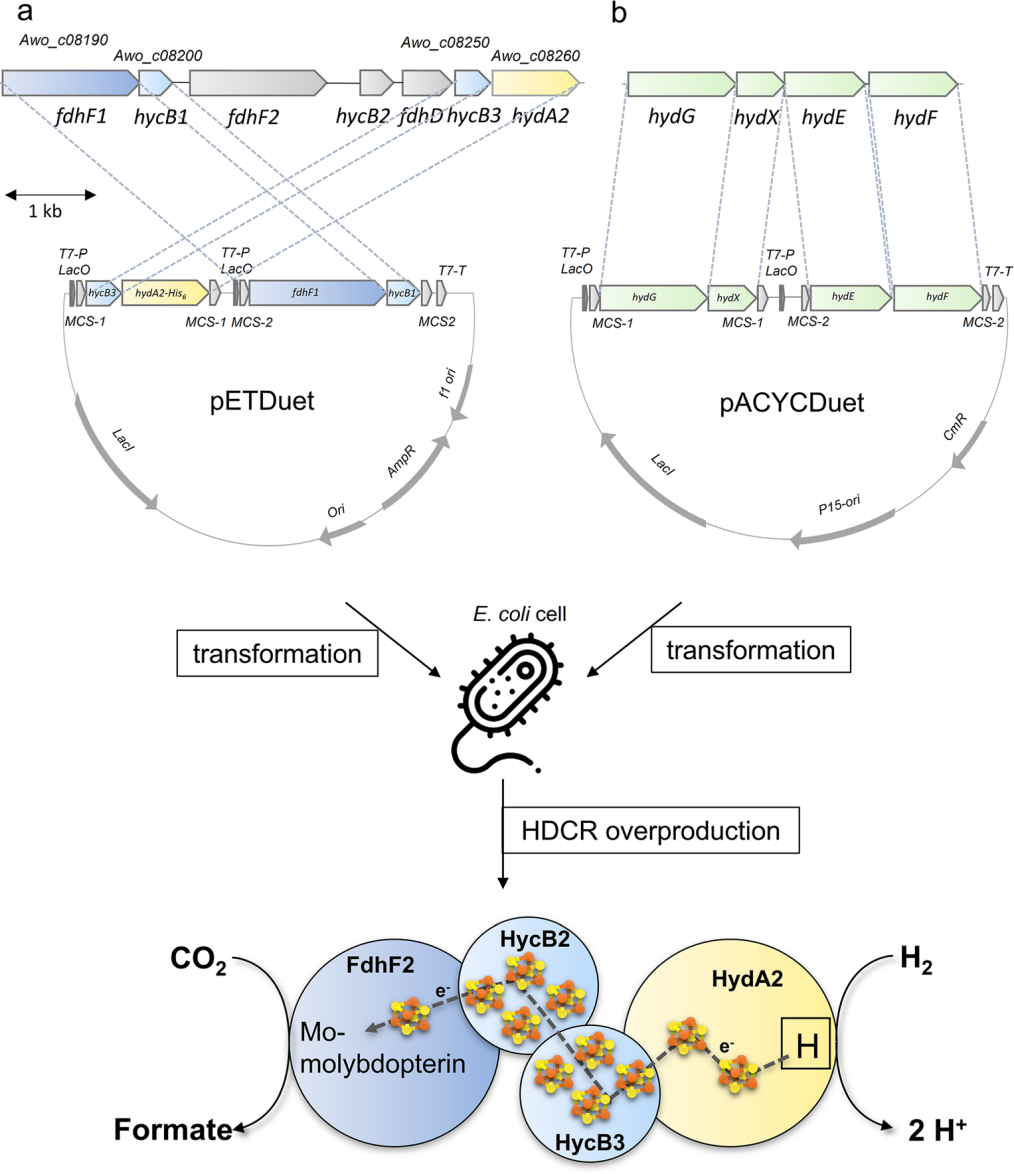
Plasmids and strategy for heterologous production of a functional HDCR complex in *E. coli*. (a) The pETDuet plasmid was used to express all four relevant HDCR genes and (b) the pACYCDuet plasmid encoded [FeFe]-hydrogenase maturases from S*. oneidensis* leading to the production of a functional HDCR complex. *HydE/F/G*, [FeFe]-hydrogenase maturases genes of S*. oneidensis; fdhF1*, formate dehydrogenase (cysteine containing) gen; *hydA2*, [FeFe]-hydrogenase gene; *hycB1/B3*, genes coding for putative electron-transferring subunits. Source: icon was designed by Freepik from www.flaticon.com

In times of global warming, the increase in atmospheric CO_2_ must be reduced and to reach the ambitious goal, carbon capture and storage (CCS) or utilization (CCU) is essential. In addition to chemical processes for CCS, biological solutions apply (Cheah et al. 2016; Kumar et al. 2018; Patel et al. 2017). Indeed, physiologically different bacteria and archaea from very different phylogenetic clades are known to reduce carbon dioxide (Berg 2011; Drake et al. 2006; Kleinsteuber et al. 2012). To date, seven pathways are known for the biological reduction of CO_2_ (Berg 2011; Fuchs 2011) and whereas all others have a demand for ATP hydrolysis the Wood-Ljungdahl pathway of CO_2_ fixation (Ljungdahl 1994; Poehlein et al. 2012; Schuchmann and Müller 2014) as catalyzed by acetogenic bacteria is energy neutral. This pathway also captures hydrogen as electron donor for CO_2_ reduction. It has two branches that each start with the reduction of CO_2_. In the carbonyl branch, CO_2_ is reduced to CO by the enzyme CO dehydrogenase/acetyl-CoA synthase (CODH/ACS) and in the methyl branch, CO_2_ is reduced to formate by a class of enzymes called formate dehydrogenases (Ljungdahl 1986; Ragsdale 2008). Formate dehydrogenases are well known from prokaryotes as well as eukaryotes (Maia et al. 2015). In most cases, the physiological function of the enzyme is to oxidize formate. Due to the low redox potential of the formate/CO_2_ couple (E^0^’ = −432mV), NAD^+^ reduction is possible and common. In contrast, formate dehydrogenases that operate in the reductive reaction need stronger reducing conditions such as a high NADPH/NADP^+^ ratio or reduced ferredoxin (Scherer and Thauer 1978; Wang et al. 2013; Yamamoto et al. 1983). Anyhow, the enzyme requires a soluble cofactor that must be reduced by hydrogen by at least one other enzyme. In contrast to the classical formate dehydrogenases, some acetogenic bacteria have a different enzyme that directly uses molecular hydrogen as reductant for CO_2_ reduction, the so-called hydrogen-dependent CO_2_ reductase (HDCR) (Schuchmann and Müller 2013; Schwarz et al. 2018). This soluble enzyme complex has been characterized from a mesophilic (*Acetobacterium woodii*) and a thermophilic (*Thermoanaerobacter kivui*) species (Schuchmann and Müller 2013; Schwarz et al. 2018). The HDCR gene cluster of *A. woodii* consists of seven genes, containing two isogenes coding for a formate dehydrogenase subunit (FdhF1, FdhF2), a [FeFe]-hydrogenase subunit (HydA2), three putative electron-transferring subunits (HycB1/B2/B3), and a putative formate dehydrogenase maturation protein (FdhD) (Schuchmann and Müller 2013) (Fig. 1a). The two variants of the formate dehydrogenase subunits only differ by the presence of cysteine (FdhF1) or selenocysteine (FdhF2) in the subunit. Both formate dehydrogenases belong to the dimethylsulfoxide reductase (DMSOR) family and harbor a molybdenum-*bis* pyranopterin guanosine dinucleotide (Mo-bisPGD) cofactor in their active site. The second catalytic subunit belongs to the class of [FeFe]-hydrogenases and has a typical [H]-cluster in its active site. Furthermore, the HDCR enzyme complex contains 12 [4Fe4S]-clusters (ISCs) which, most likely, connect the two catalytic subunits of formate dehydrogenase and hydrogenase channeling the electrons from one active site to the other (Fig. 1b) (Schuchmann and Müller 2013). The HDCRs from *T. kivui* and *A. woodii* have the highest CO_2_ reduction rates with hydrogen as reductant ever reported for a biological system and are orders of magnitude more efficient than any chemical catalyst (Müller 2019). This makes them promising candidates for biocatalysts in hydrogen storage and carbon capture. Due to the oxygen sensitivity of the enzyme, we developed a whole-cell system with both species that efficiently converts hydrogen and carbon dioxide to formate and vice versa (Kottenhahn et al. 2018; Schuchmann and Müller 2013; Schwarz and Müller 2020; Schwarz et al. 2021). The formate produced by these biocatalysts can then be fed to different formatotrophic bacteria to produce value-added compounds (Claassens et al. 2019; Hwang et al. 2020; Li et al. 2012; Yishai et al. 2016).

Recently, the industrial platform organism *Escherichia coli* was genetically engineered to use formate as carbon and energy source (Kim et al. 2020) opening the door for the production of a wide range of value-added compounds. We aimed to genetically engineer *E. coli* to produce formate from hydrogen and carbon dioxide by the HDCR from the mesophilic acetogen *A. woodii*. Since *E. coli* already encodes three Mo-*bis* PGD-containing formate dehydrogenases (Maia et al. 2015; Sawers 1994), we expected that *E. coli* contains the necessary biosynthetic pathway and maturation system for the correct incorporation of the molybdenum cofactor (Moco) into the formate dehydrogenase subunit of the HDCR. The *E. coli* proteins FdhD and FdhE which have been proposed to have a chaperone-like function and are involved in the synthesis of functional formate dehydrogenases in *E. coli* (Hartmann et al. 2014; Schlindwein et al. 1990) have similarities of 44.9% and 41.6%, respectively, to the putative formate dehydrogenase maturation protein FdhD of *A. woodii*. That FdhD from *E. coli* is capable of replacing maturation proteins with similar roles from other species was already shown for the FdsC maturation protein of the formate dehydrogenase of *Rhodobacter capsulatus* (Hartmann and Leimkühler 2013; Hartmann et al. 2014). But unlike the natural occurrence of formate dehydrogenases in the metabolism of *E. coli*, the organism does not contain any [FeFe]-hydrogenase (Forzi and Sawers 2007; Sargent 2016). This has to be taken into account, since [FeFe]-hydrogenases require accessory proteins for correct assembly and insertion of the [H]-cluster into the active site of the hydrogenase (Lubitz et al. 2014; Mulder et al. 2011). The maturase proteins HydE, HydF, and HydG seem to be conserved in all [FeFe]-hydrogenase-producing organisms (Meyer 2007). In a previous study, the successful production of functional [FeFe]-hydrogenases from *Chlamydomonas reinhardtii* and *Clostridium pasteurianum* was shown for *E. coli* by using the native maturases (HydE, HydF, HydG) of *Shewanella oneidensis* (Kuchenreuther et al. 2010). Additionally, [FeFe]-hydrogenases and the maturase proteins require FeS-cluster (Brazzolotto et al. 2006; Rubach et al. 2005). Considering all these aspects, we started out to establish the heterologous overproduction of the HDCR from *A. woodii* in *E. coli*.

## Materials and methods

### Plasmid design

To express a functional HDCR Complex, the *A. woodii fdhF1* (Awo_c08190), *hydA2* (Awo_c08260), *hycB1* (Awo_c08200), and *hycB3* (Awo_c08250) genes as well as the *hydGX* (HM357717.1, AAN56899) and *hydEF* (HM357715.1, HM357716.1) encoding the *S. oneidensis* hydrogenase maturases were cloned in two different vectors. The maturase expression construct was designed according to the procedure of Kuchenreuther et al. (2009) and was cloned on the pACYCDuet-1 vector (Novagene, Merck, Darmstadt). The genes of *A. woodii* (DSM 1030) coding for the subunits of the HDCR were cloned on a pETDuet-1 vector (Novagene, Merck, Darmstadt) and the coding sequence for a C-terminal *His-tag* extension at the hydrogenase subunit was added.

### Organisms and cultivation

For the recombinant expression in *E. coli* BL21(DE3) Δ*iscR* (Akhtar and Jones 2008) and *E. coli* JM109(DE3) (Promega, Madison, USA), the cells were transformed with the pETDuet-*HycB3-HydA2_His-FdhF1-HycB1* and the pACYCDuet-*GXEF* plasmids. For the expression of the HDCR in *E. coli* BL21(DE3) Δ*iscR*, cells were first grown aerobically in LB Miller growth medium (10 g/l tryptone; 5 g/l yeast extract; 10 g/l NaCl; 100 mM 3-(N-morpholino)propanesulfonic acid (MOPS), pH 7.4). The medium was supplemented with kanamycin (50 μg/ml), ampicillin (100 μg/ml), chloramphenicol (30 μg/ml), 25 mM glucose, 2 mM ferric ammonium citrate, and 1 mM sodium molybdate according to the procedure of Kuchenreuther et al. (2010). The non-recombinant control strain *E. coli* BL21(DE3) Δ*iscR* was handled under the same conditions. For the production of the HDCR in *E. coli* JM109(DE3), the cells were grown in ZYP medium pH 7.4 according to the procedure of Studier (2005) and supplemented in the same way as described above but without addition of kanamycin. Cultures were grown aerobically at 37 °C to an OD_600_ of 0.3–0.5 then transferred into glass flasks (Schott AG, Mainz, Germany) and switched to anaerobic metabolism by addition of 25 mM sodium fumarate and sparging with N_2_ for at least 20 min. Finally, 2 mM cysteine was added and the flasks were sealed with gas-tight rubber stoppers and cooled down to the production temperature of 16 °C. A final concentration of 1 mM Isopropyl beta-D-1-thiogalactopyranoside (IPTG) was used for the induction of the protein production for 16–24 h.

### Preparation of cell-free crude extracts and purification of the HDCR

All purification steps were performed under strictly anoxic conditions at room temperature in an anaerobic chamber (Coy Laboratory Products, Grass Lake, MI) filled with 95–98% N_2_ and 2–5% H_2_ (Heise et al. 1992). For protein purification, cultures were centrifuged at 4 °C for 10 min at 11,325×*g*, resuspended in 250 ml of buffer A (25 mM Tris, 20% [v/v] glycerol, 20 mM MgSO_4_, pH 7.5), centrifuged again, and resuspended in washing buffer A with additional 0.5 mM phenylmethylsulfonyl fluoride (PMSF) and 0.1 mg/ml DNaseI and passed through a french pressure cell press (SLM Aminco, SLM Instruments, USA) at 110 MPa. Cell debris was removed by centrifugation at 20,000×*g* for 10 min. The cleared lysate was applied to 1 ml of Ni-nitrilotriacetate (Protino 6x Histidine-tag, Macherey-Nagel GmbH & Co. KG, Düren, Germany), which was equilibrated with 15 ml of buffer 1 (150 mM 2-Amino-2-(hydroxymethyl)-1,3-propanediol (Tris), 300 mM NaCl, 20% [v/v] glycerol, 20 mM MgSO_4_, 50 mM imidazole, 4 μM resazurin, 0.5 mM dithioerythritol, pH 7.5). The resin was washed subsequently with 25 column volumes of buffer 1 and the retained protein was eluted with about 5 ml of the same buffer containing 150 mM imidazole. For further purification and analysis of the filamentous state of the purified enzyme, the elution fraction was concentrated to a final volume of about 400 μl using ultrafiltration in 100-kDa Vivaspin tubes (Sartorius Stedim Biotech GmbH, Göttingen, Germany) and applied to a Superose 6 10/300 GL prepacked column (GE Healthcare Life Sciences, Little Chalfont, UK) equilibrated with the FPLC buffer (25 mM Tris, 20mM MgSO_4_, 20% [v/v] glycerin, 300 mM NaCl, pH 7.5) and eluted at a flow rate of 0.3 ml/min.

### In vitro [FeS]-cluster assembly of the heterologous produced HDCR

[FeS]-cluster reconstitution was performed according to the method described by Tong et al. (2003). In brief, the crude extract of *E. coli* was adjusted to 2.75 mM dithioerythritol (DTE) and 2 mM cysteine. For a source of iron, 0.5 mM ferric chloride was added and 2 mM sodium sulfide nonahydrate served as a source of sulfur. The reaction mixture was incubated slightly shaking overnight at 4 °C under anoxic conditions before the cell debris was removed by centrifugation and the purification was done as described above.

### Measurement of enzyme activities

The activities of both the catalytic subunits were measured with the artificial electron acceptor methylviologen as described before (Schuchmann and Müller 2013). Measurements of methylviologen-dependent formate dehydrogenase activity were performed with formate (10 mM) as electron donor and methylviologen (10 mM) as electron acceptor in 1 ml buffer B (100 mM HEPES/NaOH, 20 mM MgSO_4_, 2 mM DTE, pH 7.0) and a gaseous phase of 100% N_2_ at a total pressure of 1.1 × 10^5^ Pa. Methylviologen reduction was monitored at 604 nm by UV/Vis spectrophotometry (*ε* = 13.9 mM^−1^ cm^−1^). Methylviologen-dependent hydrogenase activity was measured under the same conditions except that the gas phase was 100% H_2_ at a total pressure of 1.1 × 10^5^ Pa serving as electron donor and formate was omitted. For hydrogen-dependent CO_2_ reduction, 500 μg of purified enzyme was diluted in a final volume of 10 ml buffer B in 120-ml serum bottles and a gaseous phase of 80% H_2_ and 20% CO_2_ at 1 × 10^5^ Pa overpressure was applied as substrate and incubated at 30 °C in a shaking water bath. One hundred microliters aliquots of the liquid phase were withdrawn at defined time points and analyzed. Formate was measured enzymatically by using a commercially available formic acid-kit (R-Biopharm AG, Darmstadt, Germany). For hydrogen production, 500 μg of HDCR was diluted in a final volume of 1 ml buffer B in 8-ml serum bottles and was incubated in a shaking water bath at 30 °C. The gas phase was 100% N_2_ at atmospheric pressure. One hundred fifty millimolar sodium formate was used as substrate. Fifty microliters gas samples were withdrawn with a gas-tight syringe (Hamilton, Bonaduz, Switzerland). The samples were analyzed by gas chromatography using a Clarus 580 GC gas chromatograph (Perkin Elmer, Waltham, MA, USA) with a Shin Carbon ST 80/100 column (Restek GmbH, Bad Homburg, Germany) as described before (Schwarz et al. 2018).

### Cell suspension experiments

For cell suspension experiments, recombinant cells were grown in LB Miller medium as described before, harvested, and washed twice with buffer C (50 mM Tris, 20 mM MgSO_4_, 20 mM KCl, pH 7.5). Afterwards, cells were diluted to the desired concentration in a final volume of 10 ml buffer C in 120-ml serum bottles and incubated at 30 °C in a shaking water bath. For hydrogen-dependent CO_2_ reduction, the gas phase was changed to 80% H_2_ and 20% CO_2_ at 1 × 10^5^ Pa overpressure as substrate. Where needed, KHCO_3_ was added in the desired concentration. For the determination of the pH optimum, 25 mM 2-(N-morpholino) ethanesulfonic acid (MES), 25 mM MOPS, 25 mM Tris, 25 mM 2-(Cyclohexy-lamino) ethanesulfonic acid (CHES), 2 mM DTE were added to the buffer and the pH was adjusted as indicated. One hundred microliters samples of the liquid phase were withdrawn at defined time points, freed of cells by centrifugation 10,000×*g* for 5 min at 4 °C and the supernatant was analyzed for formate using a commercially available formic acid-kit (Boehringer Mannheim/R-Biopharm AG, Mannheim/Darmstadt, Germany). For hydrogen production, the cells were prepared in the same way and 150 mM sodium formate was used as substrate. The hydrogen production was measured by gas chromatography as described before (Weghoff and Müller 2016).

### Bioinformatic methods

For pairwise sequence alignment, EMBOSS Water was used with default settings (Chojnacki et al. 2017). All amino acid sequences were retrieved from the Uniprot database. All DNA sequences were retrieved from the National Center for Biotechnology Information database.

### Analytical methods

Protein concentration was measured according to Bradford (1976). Proteins were separated in 12% polyacrylamide gels and stained with Coomassie brilliant blue G250 (Weber and Osborne 1969). LC-MS/MS and ICP-MS were performed by commercial suppliers. Protein concentration of whole cells for the cell suspension experiments was measured according to Schmidt et al. (1963).

### Statistics and reproducibility

The number of replicates and types of replicates which were performed are described in the legend of each figure. The mean of individual data points is shown with its standard deviation (± SD); this information is provided in each figure legend. No statistical tests were needed or performed.

## Results

### Overproduction and molecular analysis of the HDCR

**Fig. 2:**
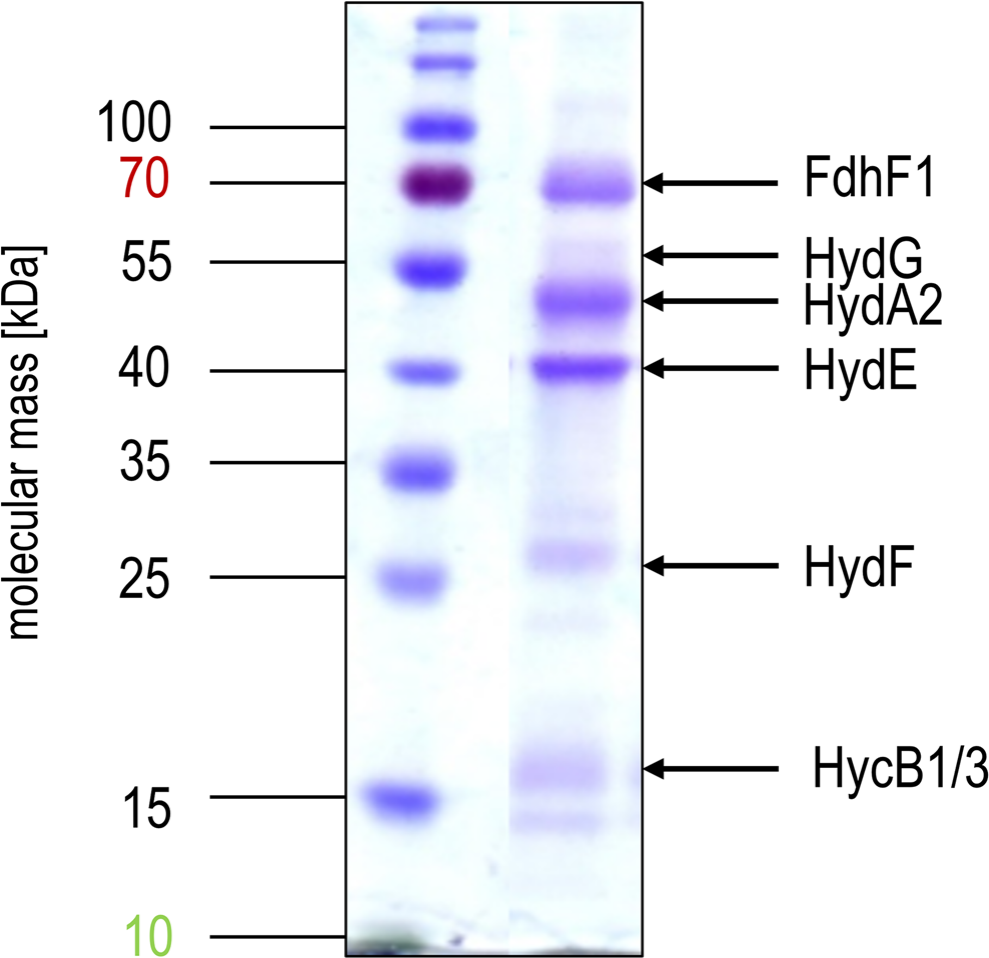
Purified HDCR complex from *E. coli BL21*(DE3) *ΔiscR*. 10 μg purified HDCR was separated on a 12% SDS-PAGE stained with Coomassie-Brillant Blue (right lane). The left lane contains the molecular mass standard. Proteins were identified by peptide mass fingerprinting

**Fig. 3:**
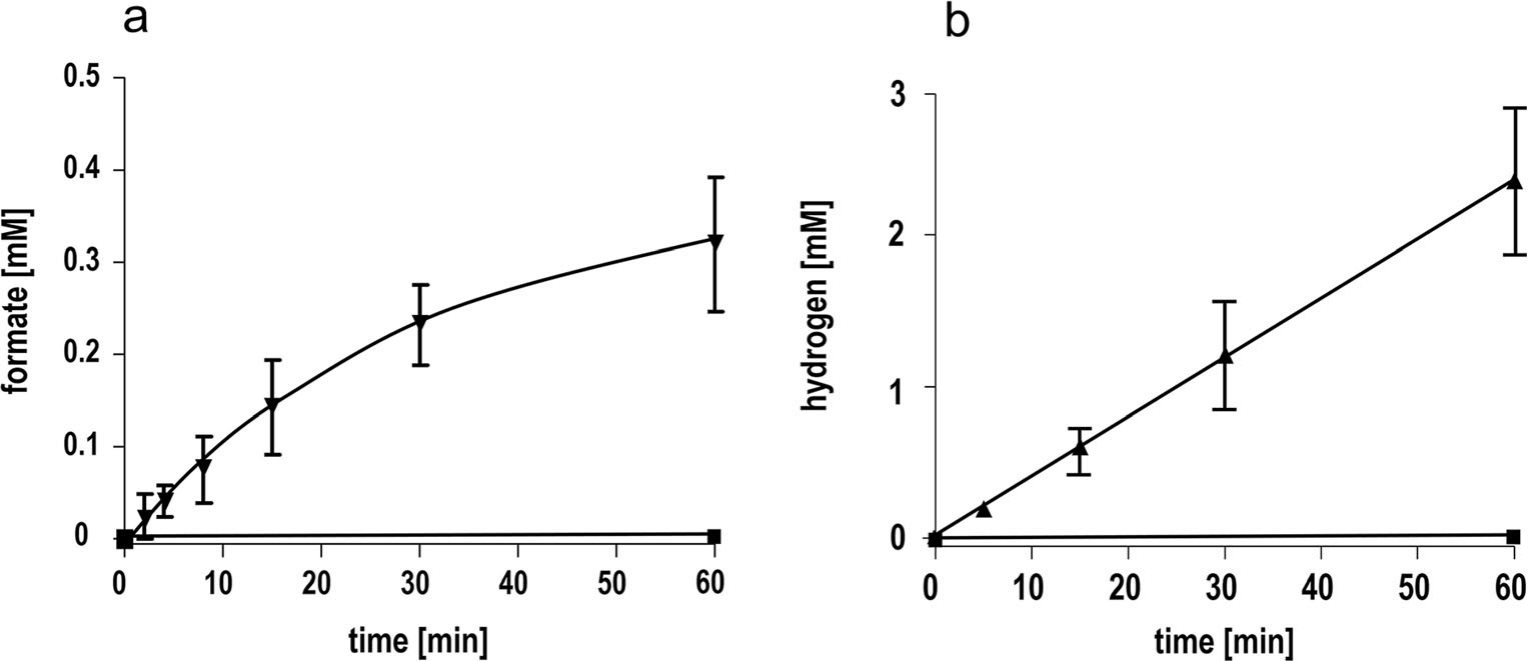
Formation of formate or hydrogen by the purified HDCR produced in *E. coli* BL21(DE3) *ΔiscR*. (a) 600 μg purified HDCR (triangles down) and buffer containing no enzyme (squares) were incubated with H_2_ + CO_2_ (80:20%, 1 × 10^5^ Pa overpressure). Formate formation was quantified enzymatically. (b) 600 μg purified HDCR (triangles up) and buffer containing no enzyme (squares) were incubated with 150 mM sodium formate. Hydrogen formation was determined via gas chromatography. All data points are mean ± SD and measurements were taken from distinct samples (*n* = 2)

**Fig. 4:**
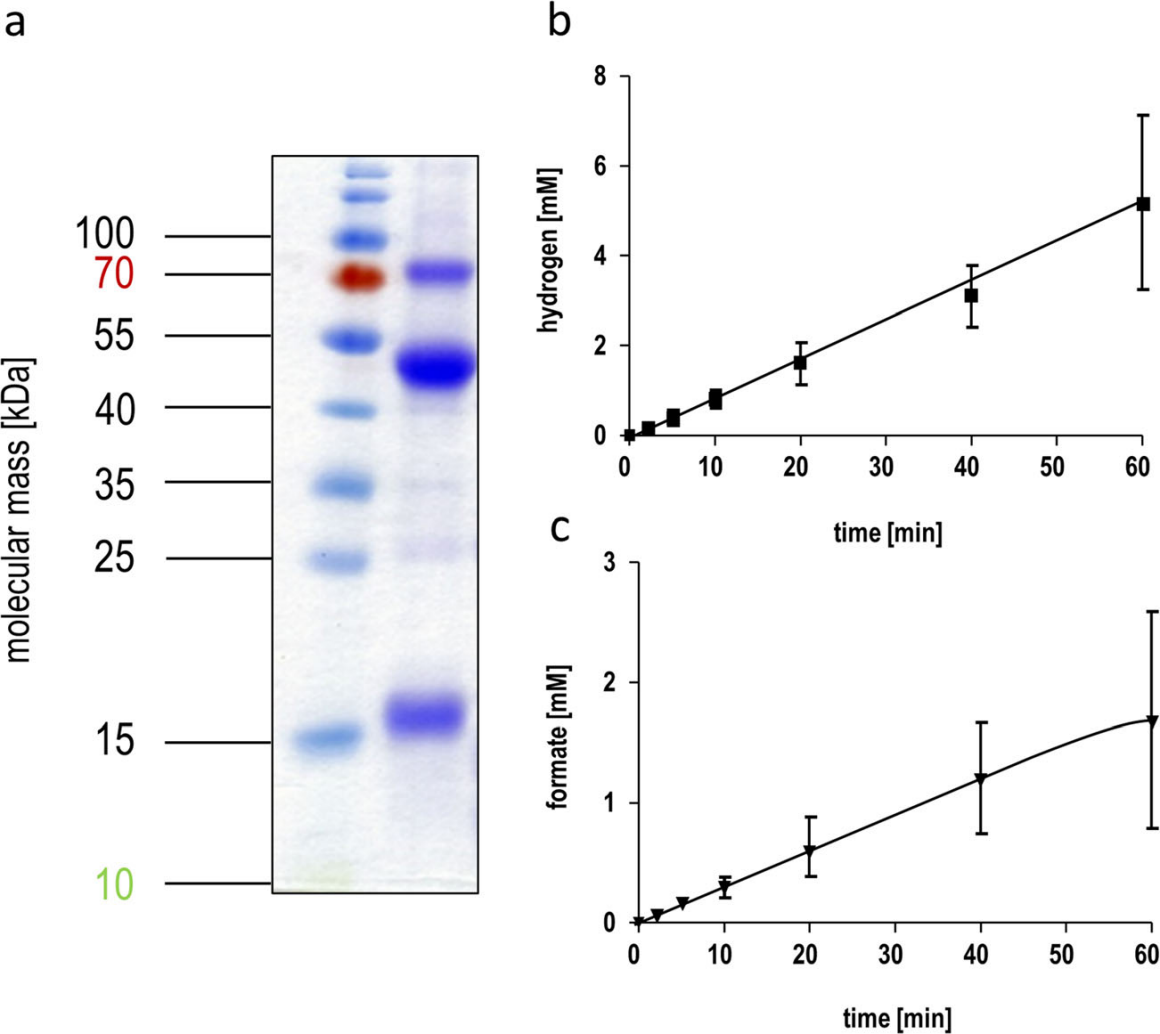
Purified HDCR complex from *E. coli* JM109(DE3). (a) 10 μg purified HDCR was separated on a 12% SDS-PAGE stained with Coomassie-Brillant Blue. (b) Formate-driven H_2_ production and (c) hydrogen-dependent CO_2_ reduction catalyzed by the purified enzyme. 0.5 mg purified HDCR was used for both reactions. All data points are mean ± SD and measurements were taken from distinct samples (*n* = 2)

The HDCR genes *fdhF1*, *hydA2*, *hycB3*, and *hycB1* were cloned in the plasmid pETDuet under the control of a T7 promotor (Fig. 1a). A sequence encoding a hexa-histidin tag was added at the 3′-end of *hydA2* to enable purification of the protein via affinity chromatography. Additional to the HDCR genes, the hydrogenase maturation genes *hydG/X/E/F* of *S. oneidensis* were cloned in the vector pACYCDuet (Fig. 1b). Both plasmids were transformed in the FeS-cluster-overproducing strain *E. coli* BL21(DE3) ∆*iscR* (Akhtar and Jones 2008) and expression was performed under anoxic conditions according to the protocol of Kuchenreuther et al. (2010). Therefore, the organism was initially grown aerobically in LB Miller medium to an optical density of 0.3–0.5 in the presence of glucose (25 mM), Na_2_MoO_4_ (1 mM), and ammonium ferric citrate (2 mM). Then, the culture was shifted to anoxic conditions by flushing the medium with N_2_-gas (100%) and the production phase of the HDCR was initiated by adding IPTG to the culture. Added fumarate (25 mM) served as the terminal electron acceptor for anaerobic respiration. After a production time of 16–24 h, the cells were harvested and disrupted via a French pressure cell press and the cell debris was removed by centrifugation. The cell-free extract was applied to a Ni-NTA column, and proteins were eluted by imidazol. The preparation as analyzed by SDS-PAGE contained six proteins and LC-MS/MS identified the subunits FdhF1, HydA2, HycB1, and HycB3, indicating the production of all four anticipated subunits (Fig. 2). In addition, the maturase proteins HydE, HydF, and HydG were present and the 60, 30, and 43 kDa proteins were identified as HydG (54.1 kDa), HydE (40.3 kDa), and HydF (43 kDa), respectively. Furthermore, the purified HDCR enzyme complex from *E. coli* was catalytically active and showed a formate production activity (H_2_:CO_2_-oxidoreductase activity) of 0.14 U/mg (Fig. 3a). Notably, the reverse reaction, formate oxidation to H_2_ and CO_2_ (formate:H^+^-oxidoreductase activity) was also catalyzed with a specific activity of 0.67 U/mg (Fig. 3b). Gelfiltration of the purified enzyme revealed the expected quaternary structure in filaments, since the purified enzyme complex showed a molecular mass bigger than 3 MDa (Figure S1). The impact of filamentation of the HDCR on the catalytic activity was already shown for the HDCR purified from *A. woodii* demonstrating that activity was reduced by 55% in the depolymerized state (Schuchmann et al. 2016). These results clearly demonstrate that a functional HDCR complex can be produced in *E. coli*. However, the molybdenum (as well as tungsten) content of the purified HDCR as determined by ICP-MS analysis was only 0.05 mol molybdenum per mol HDCR which is consistent with a low CO_2_ reduction as well as formate oxidation activity. The reason for this is most likely related to the used production strain which has major defects in anaerobic metabolism, metal ion transport, and metalloprotein biosynthesis (Pinske et al. 2011). An insufficient over production of formate dehydrogenases from different organisms has also been reported for *E. coli* BL21(DE3) (Alissandratos et al. 2014). In consequence, we switched to *E. coli* JM109(DE3) which also allows T-7 based gene expression and has no reported genotypical deficiencies in molybdenum cofactor biosynthesis and incorporation but misses the deletion of the *iscR* gene (Yanisch-Perron et al. 1985). The media of the new host organism was adjusted and the HDCR was overproduced and purified as described before. Again, the SDS-PAGE revealed the six proteins (Fig. 4a) and the molybdenum content of the purified HDCR complex determined by ICP-MS was 0.7 mol molybdenum per mol HDCR. As expected, H_2_:CO_2_-oxidoreductase (0.53 U/mg) and formate:H^+^-oxidoreductase activities (1.4 U/mg) (Fig. 4b) were strongly increased (280% and 110%, respectively) but still an order of magnitude lower compared to the enzyme purified from *A. woodii* (10 and 14 U/mg) (Schuchmann and Müller 2013). Since the HDCR complex harbors many [4Fe4S]-clusters, responsible for an efficient electron transport within the enzyme, the missing *iscR* gene deletion is most likely the reason for the low HDCR activity. The *iscR* gene encodes a transcriptional regulator (IscR) which represses the expression of genes encoding the proteins for FeS-cluster assembly. It was previously shown that its deletion resulted in an enhanced expression of the *isc* operon, leading to an increasing amount of accessible FeS-clusters within the cell (Schwartz et al. 2001). Additionally, *iscR* deletion stimulated recombinant [FeFe]-hydrogenase activity in *E. coli* (Akhtar and Jones 2008). To eliminate the burden of [FeS]-clusters assembly, the [FeS]-clusters were reconstituted in cell-free extracts of *E. coli* JM109(DE3). Therefore, the HDCR was overproduced as described and the [FeS]-cluster reconstitution was performed for 16 h at 4 °C in the cell-free extract. Afterwards, the enzyme was purified and the catalytic activities of CO_2_-reduction and formate oxidation were determined. Impressively, the H_2_:CO_2_-oxidoreductase and formate:H^+^-oxidoreductase activity increased again by a factor of 17.5 and 4.6, respectively. The enzyme catalyzed the hydrogen-dependent CO_2_ reduction with a specific activity of 9.3 U/mg and the reverse reaction was catalyzed with an activity of 6.47 U/mg, which is 93% and 46%, respectively, of the HDCR activity isolated from *A. woodii*. ICP-MS revealed a Mo and Fe content of 0.66 mol molybdenum per mol HDCR and 46 mol iron per mol HDCR which is consistent with the numbers of the enzyme purified from *A. woodii* (Schuchmann and Müller 2013). The total amount of overproduced HDCR was 1 mg purified enzyme per liter of cultivation broth for *E. coli* BL21(DE3) Δ*iscR* and 0.4 mg per liter cultivation for *E. coli* JM109(DE3) which is 7- to 10-fold more than the yield from *A. woodii*.

### Whole-cell biocatalysis for H_2_-dependent CO_2_ reduction

**Fig. 5:**
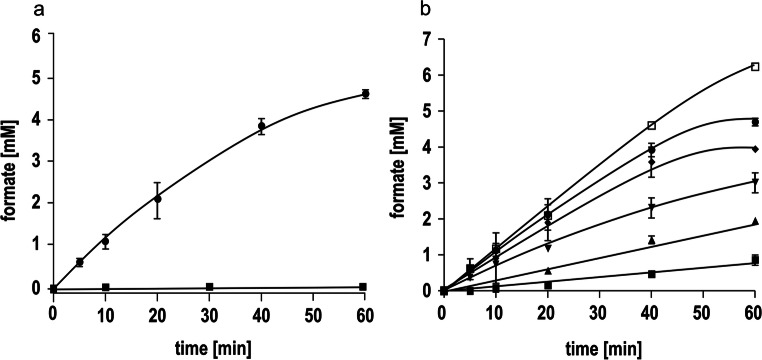
Production of formate using whole-cell catalysis. (a) Whole cells of non-recombinant (square, 30 mg/ml) and recombinant *E. coli* BL21(DE3) *ΔiscR* cells (circles, 15 mg/ml) were incubated with H_2_ + CO_2_ (80:20%, 1 × 10^5^ Pa overpressure). Non-recombinant *E. coli* cells cover *E. coli* BL21(DE3) *ΔiscR* without additional plasmids whereas recombinant *E. coli* cells cover *E. coli* BL21(DE3) *ΔiscR* plus HDCR and maturation plasmids. (b) Different cell concentrations of recombinant *E. coli* BL21(DE3) *ΔiscR* cells were incubated with H_2_ + CO_2_ (80:20%, 1 × 10^5^ Pa overpressure). Squares, 1 mg/ml; triangles up, 2 mg/ml; triangles down, 5mg/ml; diamonds, 10 mg/ml; dots, 15 mg/ml; squares empty, 30 mg/ml. All data points are mean ± SD and measurements were taken from distinct samples (*n* = 2)

Next, we aimed to establish a recombinant *E. coli* whole-cell biocatalyst to convert H_2_ and CO_2_ into formate and vice versa. Since in *E. coli* strain BL21(DE3) the genes *modABC/E/F* and *fnr* are deleted and the genes encoding Hyd-1/2/3 and FdhD/E carry mutations, many relevant enzymes of the natural hydrogen and formate metabolism such as [NiFe]-hydrogenases and molybdenum-containing formate dehydrogenases totally lack activity (Pinske et al. 2011). Therefore, *E. coli* strain BL21(DE3) ∆*iscR* was chosen as background. *E. coli* JM109 (DE3) was not considered suitable since no defects in the formate hydrogen-lyase system were reported. The BL21(DE3) ∆*iscR* strain was grown in LB Miller complex medium and resting cells were prepared. As expected, the addition of H_2_ + CO_2_ (80:20 [v/v], 1 bar overpressure) to the cell suspension resulted in the production of formate with a rate of 0.119 mmol g_CDW_^−1^ h^−1^ (0.238 mmol g^−1^ h^−1^) (Fig. 5a). An increase in the final cell concentration increased the amount of formate produced (Fig. 5b). At a cell concentration of 15 mg/ml, 4.66 mM formate was produced after 1 h. The control strain without the plasmids could neither convert H_2_ and CO_2_ to formate nor oxidize formate. As shown by Jo and Cha (2015), the native FHL-pathway of formate dissimilation to H_2_ and CO_2_ can be reactivated by the heterologous production of uptake [NiFe]-hydrogenases. To exclude any cross-reaction of our system with the native FHL-system of *E. coli*, the hydrogenase subunit of the HDCR HydA2 and its corresponding putative electron-transferring subunit hycB3 were solely produced in *E. coli* BL21(DE3) *ΔiscR*. No formate-based hydrogen production but hydrogen-dependent methylviologen reduction in cell-free extracts of recombinant cells was observed (data not shown) whereas the non-recombinant strain showed no hydrogenase activity at all, leading to the conclusion that the heterologous expression of the HDCR module expanded the ability of *E. coli* BL21(DE3) ∆*iscR* to convert H_2_ and CO_2_ into formate without the need of any external co-factors or endogenous enzymes in the reaction process.

### Biotechnologically important parameters

**Fig 6:**
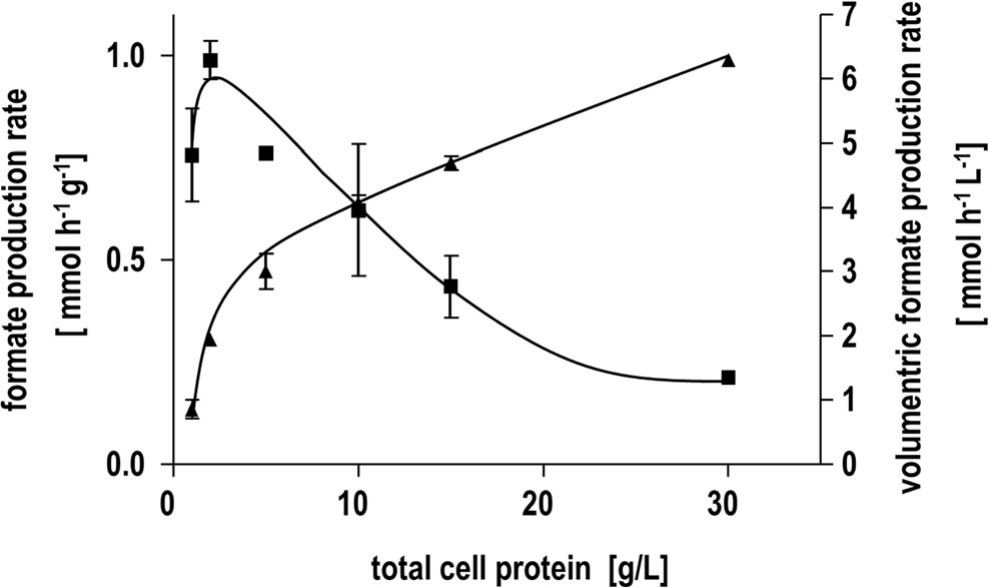
Specific and volumetric formate production rates for recombinant *E. coli* BL21(DE3) *ΔiscR* producing HDCR. Different concentrations of recombinant *E. coli* BL21(DE3) *ΔiscR* cells were incubated with H_2_ + CO_2_ (80:20%, 1 × 10^5^ Pa overpressure) leading to different specific and volumetric formate production rates. The increase of total cell protein resulted in an increased volumetric formate production rate and in a decrease of the specific formate production rate. All data points are mean ± SD and measurements were taken from distinct samples (*n* = 2)

After the proof of principle, we determined the specific formate production rate and the volumetric formate production rate (Fig. 6). At a cell concentration of 2 mg/ml, the highest specific formate production rate of 0.5 mmol g_CDW_^−1^ h^−1^ (1 mmol g^−1^ h^−1^) was observed. Increasing cell densities resulted in a linear increase of the volumetric formate production rates up to 6 mmol L^−^^1^ h^−1^ at 30 mg ml^−1^ with a simultaneous decrease in the specific rates. To further increase the specific formate production rate of the recombinant *E. coli* BL21(DE3) ∆*iscR* cells, additional potassium bicarbonate was added. The added bicarbonate should lead to a higher concentration of dissolved CO_2_ through the interconversion of bicarbonate to CO_2_ by the native carbonic anhydrase of *E. coli* (Guilloton et al. 1992)*.* The addition of bicarbonate to the resting cells clearly increased the specific formate production rates by a factor of around 4 to a maximum rate of 0.4 mmol g_CDW_^−1^ h^−1^ at 5 mg/ml total cell protein and with additional 250 mM bicarbonate (Figure S2). The impact of pH on *E. coli* cells for H_2_-dependent CO_2_ reduction was also investigated. As expected, a more alkaline pH favored the formation of formate from H_2_ and CO_2_ compared to acidic prevailing conditions. Up to pH 8, an increase in the specific formate formation rates to 0.39 mmol g_CDW_^−1^ h^−1^ (0.78 mmol g^−1^ h^−1^) was observed until the rates dropped by ~53% at pH 10 (Figure S3).

## Discussion

Recombinant *E. coli* strains were already designed for increased endogenous CO_2_ recycling (Lee et al. 2020) as well as for the direct hydrogenation of CO_2_ to formate (Alissandratos et al. 2014; Roger et al. 2018). In this study, we have shown for the first time that the biotechnologically interesting enzyme complex HDCR can be heterologously produced in *E. coli* with high activity. The HDCR operates independent of the *E. coli* metabolism and cross-reactions of the produced hydrogenase with the native FHL-system of *E. coli* BL21(DE3) were experimentally excluded. The data are consistent with the studies of Jo and Cha (2015) which clearly showed that a reactivation of the formate hydrogen-lyase system by the heterologous expression of three [NiFe]-hydrogenases is dependent on a signal peptide of the small subunits of the hydrogenases for membrane interaction. Since the HDCR is a soluble enzyme, the [FeFe]-hydrogenase subunit lacks that kind of signal peptide. In a previous and so far only study, in which recombinant *E. coli* JM109(DE3) cells were used for hydrogen-dependent CO_2_ reduction to formate (Alissandratos et al. 2014), the overall system was dependent on the catalytic activity of one or more of the endogenous hydrogenases of *E. coli*. The study showed that the overexpression of formate dehydrogenase genes from *Clostridium carboxyvorans*, *Pyrococcus furiosus*, or *Methanobacterium thermoformicum* enabled *E. coli* JM109(DE3) for the production of formate from H_2_ and CO_2_, the latter in form of bicarbonate. The recombinant *E. coli* JM109(DE3) cells had a specific formate production rate of around 0.1 mmol g_CDW_^−1^ h^−1^ which is 4 times lower than the rates observed in this study (Table 1). In addition, our HDCR-based system has a great potential for future upgrading, for example, the replacement of cysteine by selenocysteine in the formate dehydrogenase subunit of the HDCR. It was shown for *E. coli* that the formate dehydrogenase activities were decreased dramatically by two orders of magnitude if cysteine instead of selenocysteine was incorporated into the formate dehydrogenase H (Axley et al. 1991). Here, we decided to use the selenocysteine-free formate dehydrogenase (FdhF1) instead of the selenocysteine containing formate dehydrogenase gene (FdhF2) since the overexpression of *fdhF2* resulted in a truncated version of the coding enzyme in *E. coli* (data not shown). The incorporation of selenocysteine instead of cysteine occurs co-translationally and requires a complex machinery consisting of three different proteins (SelA/B/D), a specific tRNA^Sec^ (SelC) and a secondary structure in the selenoprotein-encoding mRNA, called Sec Insertion Sequence element (SECIS element) (Böck et al. 1991; Gonzalez-Flores et al. 2013). Nevertheless, different approaches were already shown to achieve the incorporation of selenocysteine in heterologous produced proteins in *E. coli* (Arner et al. 1999; Chen et al. 1992; Heider and Böck 1992; Rengby et al. 2004) and could be one likely way to further increase the activity of the heterologous overproduced HDCR up to the “original” HDCR activity of *A. woodii*.

**Table 1:** Comparison of different genetically modified *E. coli* strains for hydrogen-dependent CO_2_ reduction to formate under various conditions

**Organism**	**Reaction condition: temperature [°C]**	**Reaction condition: over pressure [bar]**	**Reaction condition: pH**	**Mode**	**Specific formate production rate [mmol g**_**CDW**_^**−1**^**h**^**−1**^**]**	**Ref.**
*E. coli* BL21(DE3) *ΔiscR*	30	1	7.5	Closed-batch (flasks)	**0**	This study
***E. coli*** **BL21(DE3)** ***ΔiscR*** **rec. strain** **(*pHDCR pGXEF)***	30	1	7.5	Closed-batch (flasks)	**0.115 ±0.025**	This study
***E. coli*** **BL21(DE3)** ***ΔiscR*** **rec. strain** ***(pHDCR pGXEF)***	30	1 **+ 250 mM potassium bicarbonate**	7.5	Closed-batch (flasks)	**0.40 ±0.06**	This study
*E. coli* K-12 FTD89 *(ΔhyaB, ΔhybC)*	37	~0.4	7.4	Closed-batch (Hungate tubes)	**~0.41**	Roger et al. (2018)
*E. coli* K-12 RT1 *(ΔhyaB, ΔhybC, ΔpflA, ΔfdhE)*	37	10	8	Closed-batch bioreactor, pH-controlled	**~15**	Roger et al. (2018)
*E. coli JM109* *rec. strain* ^a^	37	0 **+ 250 mM sodium bicarbonate**	7.0	Closed-batch (flasks)	**~0.1**	Alissandratos et al. (2014)

^a^Recombinant *E. coli* strain JM109(DE3) overexpressing *fdh* of *Pyrococcus furiosus* (FDH_Pyrfu)

Another possibility to increase the catalytic rates and to achieve a hydrogen-dependent CO_2_ reduction in *E. coli* is the use of an optimized reactor system to change the prevailing environmental conditions and to shift the chemical equilibrium to the side of the product formate. The directionality of the membrane-bound formate hydrogenlyase (FHL) complex of *E. coli* is strongly affected by pH and partial pressure in the system (Roger et al. 2018). FHL normally favors the disproportionation of formate to H_2_ and CO_2_ in nature. But in a highly pressurized (up to 10 bar overpressure) and pH-controlled bioreactor system, the FHL was unlocked in the “reverse reaction” functioning as a hydrogen-dependent CO_2_ reductase for the direct hydrogenation of CO_2_ to formate. Modified *E. coli* K-12 cells reached specific formate formation rates of around 15 mmol g_CDW_^−1^ h^−1^ and final formate titers of ~500 mM (Roger et al. 2018). The combination of such a highly pressurized bioreactor system with an optimized recombinant *E. coli* system would therefore be a promising and interesting approach for future applications in *E. coli*–based CO_2_ conversion to formate.

In sum, this study demonstrates the transformation of *E. coli* BL21(DE3) ∆*iscR* cells into microbial cell factories for the conversion of H_2_ + CO_2_ into formate even under moderate reaction conditions (1 bar overpressure, 30 °C). We could further increase the specific formate formation rates to 0.4 mmol/g_CDW_*h by adding bicarbonate, rates which were so far never reported for an implemented CO_2_ reduction system in *E. coli*. Besides, the study clearly points out the great potential of whole-cell biocatalysis for H_2_-dependent CO_2_ reduction in *E. coli* using a future FHL-, hydrogenases-, and *iscR*-deficient strain. Many chemical catalysts suffer from low turnover frequencies (TOF), dependence on high pressure or temperature or even very expensive additives, emphasizing the biological route for H_2_-storage and CO_2_-capturing by using the easy handable platform organism *E. coli*. Together with the recently described formatotrophic strain of *E. coli* (Kim et al. 2020; Yishai et al. 2018) this could pave the road to make *E. coli* a chemolithoautotrophic bacterium.

## Supplementary information

ESM 1 (PDF 221 kb)

## Data Availability

All datasets and material generated or analyzed in this study are available from the corresponding author upon reasonable request.
