# Supplementary material for: Capture of carbon dioxide and hydrogen by engineered Escherichia coli: hydrogen-dependent CO2 reduction to formate
Source: Appl Microbiol Biotechnol. 2021 Jul 31;105(14-15):5861–72. doi: 10.1007/s00253-021-11463-z (PMC8390402; doi:10.1007/s00253-021-11463-z)
Supplement: Supplementary file 1 — (PDF 221 kb) [file 253_2021_11463_MOESM1_ESM.pdf]

SUPPLEMENTARY MATERIAL

Journal: Applied Microbiology and Biotechnology

# Capture of carbon dioxide and hydrogen by engineered *Escherichia coli*: hydrogen-dependent $CO_2$ reduction to formate

Felix Leo[1], Fabian M. Schwarz[1], Kai Schuchmann[1], Volker Müller[1]*

[1]Molecular Microbiology & Bioenergetics, Institute of Molecular Biosciences, Johann Wolfgang Goethe University, Frankfurt am Main, Germany

[*]Corresponding author: Volker Müller, phone +496979829507, fax +496979829306, mail: vmueller@bio.uni-frankfurt.de

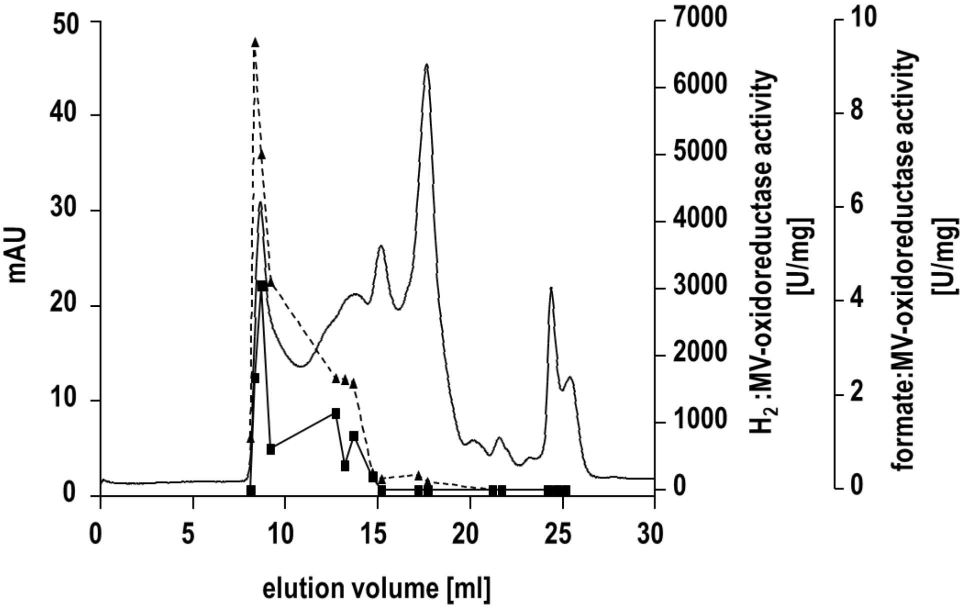


**Figure S1. Gel filtration of the purified HDCR complex.** 616 µg $Ni^{2+}$-NTA purified HDCR produced in *E. coli* BL21(DE3) *ΔiscR* were separated on a Superose 6 10/300 GL prepacked gelfiltration column under anoxic conditions. The majority of HDCR activity was found in the void volume revealing a molecular mass >5 MDa. The artificial electron acceptor methylviologen was used to detect the catalytic subunits. $H_2$:methylviologen-oxidoreductase activity (triangles) and formate:methylviologen-oxidoreductase activity (squares) was mainly measured in the void volume.

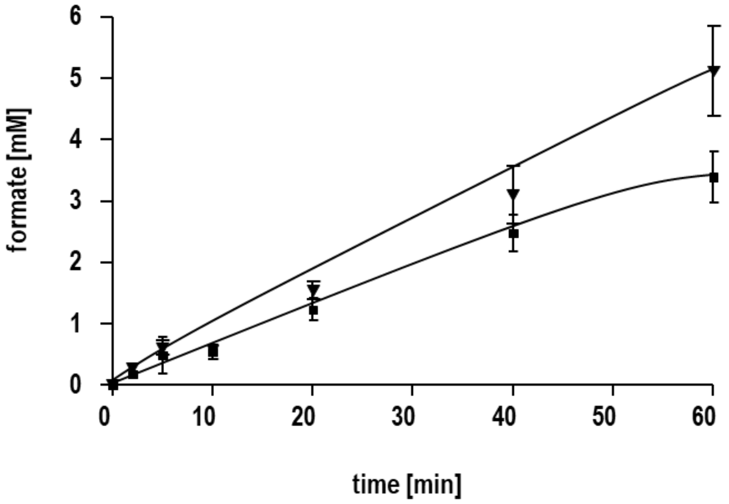


**Figure S2. Effect of potassium bicarbonate on formate formation by recombinant *E. coli* BL21(DE3) *ΔiscR* whole cells with $H_2$ + $CO_2$ as substrate.** Resting cells of recombinant *E. coli* JM109(DE3) (5 mg/ml) were incubated with $H_2$ + $CO_2$ (80:20%, $1 \times 10^5$ Pa overpressure) without $KHCO_3$ (squares) and 250 mM $KHCO_3$ (triangles).

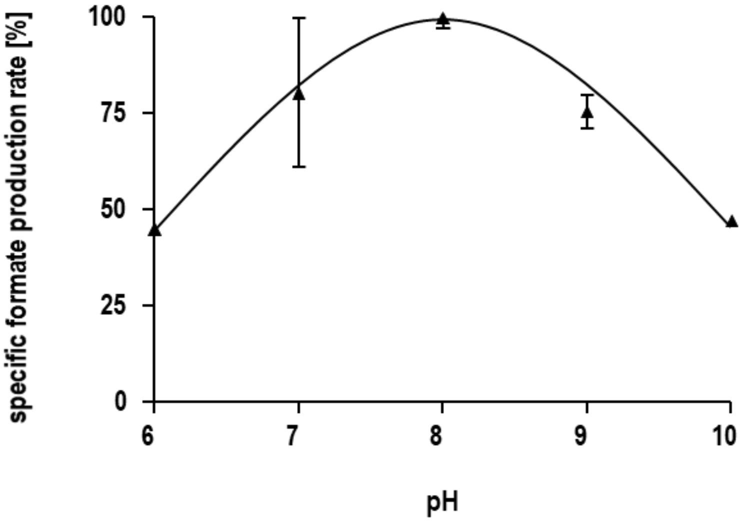


**Figure S3. pH profile of hydrogen-dependent carbon dioxide reduction by whole cells of *E. coli* BL21(DE3) *ΔiscR* producing HDCR.** Shown is the pH profile for the specific formate production at the given pH using cells of *E. coli BL21 DE3 ΔiscR* (5mg/ml) incubated with $H_2$ + $CO_2$ (80:20%, 1 × $10^5$ Pa overpressure). 100% of the activity correspond to 0.39 mmol $g_{CDW}^{-1}$ $h^{-1}$. All data points are mean ± SD, N = 2.
